# The Effect of Epidural Bupivacaine on BIS Levels in the Awake Phase and on the Maintenance Doses of Propofol and Fentanyl During General Anesthesia

**DOI:** 10.5812/aapm.5461

**Published:** 2013-03-26

**Authors:** Reza Shariat Moharari, Afshin Samadi, Farsad Imani, Mahdi PanahKhahi, Patricia Khashayar, Alipasha Meysamie, Atabak Najafi

**Affiliations:** 1 Department of Anesthesiology, Tehran University of Medical Sciences, Tehran, Iran; 2 Department of Community Medicine, Tehran University of Medical Sciences, Tehran, Iran

**Keywords:** Anesthesia, Epidural, Bupivacaine

## Abstract

**Background:**

Simultaneous administration of epidural local anesthetic agents (LA) and general anesthetics (intravenous or inhaled) is a common procedure in patients undergoing major operations. The effects of epidural anesthesia during combined general-epidural anesthesia on the alertness level (CGEA) in the awake phase and the doses of anesthetics have been reported.

**Objectives:**

The present study was designed to determine the effects of epidural bupivacaine on the alertness level measured by bispectral index (BIS) in the awake phase and the maintenance doses of propofol and fentanyl during general anesthesia for vascular operation on the lower limb.

**Patients and Methods:**

A double-blinded randomized clinical trial was conducted on patients awaiting vascular surgery on lower extremities in a teaching hospital from October 2007 to October 2008. During the epidural anesthesia, the control group received 0.9% NS while 0.125% bupivacaine was injected in the case group via the epidural route. No sedative drug was utilized for epidural catheter placement. The BIS measurement was performed in both groups during the awake phase, before performing epidural anesthesia, and 10 minutes after epidural injection at 1-min intervals for 15 min. After induction of general anesthesia in both groups, anesthesia maintenance was established using the infusion of propofol with the aim of keeping the BIS level between 40 and 50 throughout the anesthesia. At the end of the study period, maintenance dose requirements of propofol and fentanyl were measured.

**Results:**

Thirty-two patients were enrolled in the study. There was no difference in BIS levels of the two groups in the awake phase. There was a significant difference between the propofol and fentanyl requirements of the two groups.

**Conclusions:**

Performing CGEA using bupivacaine was reported to reduce propofol and fentanyl doses required to maintain BIS levels between 40 and 50 considerably.

## 1. Background 

Simultaneous administration of epidural local anesthetic agents (LA) and general anesthetics (intravenous or inhaled) is a common procedure in patients undergoing major operations (1). Such concurrency, also known as combined general-epidural anesthesia (CGEA), has several advantages including early recovery from general anesthesia and postoperative analgesia along with reduced risk of cardiac dysrhythmia, deep vein thrombosis and other cardiovascular and ischemic events; as a result, this method is commonly used in certain centers all around the world (2). Several side effects such as local anesthetics dose-related hypotension as well as bradycardia and respiratory distress related to the site of regional anesthesia (RA), and light anesthesia leading to awareness during the operation have been reported following this method too. In different centers, various concentrations of different LAs are used, while general anesthesia is administered empirically or based on cardiovascular responses during the surgery without knowing the depth of anesthesia. Several studies have shown that some drugs can be used instead of opioids and anesthetics (3-6), and have also evaluated the effects of different LA agents on anesthetic requirements during CGEA using bispectral index (BIS) monitoring; the optimal combination and dose, however, have not been yet reported (4, 5, 7, 8).

## 2. Objectives

Bupivacaine is the commonly used LA in the center that subjects referred to; therefore the current study aimed to determine the effects of this medication on the alertness level (BIS) in the awake phase, and the maintenance doses of propofol and fentanyl in general anesthesia.

## 3. Patients and Methods

After being approved by ethical board committee of Tehran University of Medical Sciences (TUMS), a double-blinded randomized clinical parallel trial was conducted on patients awaiting vascular surgery on lower extremities in a teaching hospital from October 2007 to October 2008. All the selected patients had an ASA (The American Society of Anesthesiologists) physical status of I or II. Those suffering from underlying neurologic diseases, bleeding and coagulation disorders, and severe lumbar alignment disorders along with those addicted to drugs or alcohol, and individuals with previous history of hypersensitivity reaction to local anesthetics were excluded from the study. An informed written consent was obtained from all the patients. Thereafter, they were divided into two groups using the random allocation software (9). The allocation ratio was 1:1. All patients were preloaded with 10 mL/kg normal saline (NS). Following the injection of 2cc lidocaine 1% (local), an epidural catheter was placed between L4-L5, through an 18 gauge Tuohy needle using a loss of resistance technique, while the patient was sitting. No sedative drug was utilized for epidural catheter placement. Thereafter, the control group received 8 mL of 0.9% NS while 8 mL of 0.125% bupivacaine was injected (10) in the case group as a bolus via the epidural route 25 min before induction of anesthesia, then based on the group allocation, infusion was maintained at 8 mL/h through the syringe pump (Model SP-100 s, JMS, Hiroshima, Japan). The anesthesiologist performing the epidural block and setting the epidural infusion was not aware of the group allocation; he administered the epidural injections prepared by another independent anesthesiologist unaware of the objectives of the study and what he is injecting. Sensory block was evaluated at 10 and 20 minutes after the epidural injection using pinprick; cephalad sensory block at T10 level was considered acceptable. Intra-operative monitoring consisted of 5 lead electrocardiography, heart rate (HR), Non-invasive measurement of blood pressure (NIBP), invasive arterial blood pressure, end-tidal carbon dioxide, pulse oximetry, temperature, BIS (CSM, Danmeter A/S, Denmark), and urine output. The BIS measurement was performed during the awake phase, before performing epidural anesthesia and 10 minutes after epidural injection at 1-min intervals for 15 min. Induction of anesthesia was done with IV fentanyl (Hexal, Germany) 2 µg/kg, IV midazolam (Daroopakhsh, Iran) 0.04 mg/kg, IV propofol (B. Braun, Germany) 1.5 mg/kg, and IV atracurium (Aboureyhan, Iran) 0.5 mg/kg. Ventilation was controlled (Julian, Dragerwerk, Lubeck, Germany) with a tidal volume of 10 mL/kg and respiratory rate was adjusted to maintain end-tidal carbon dioxide between 30–35 mm Hg. Maintenance of anesthesia was isoflurane. After endotracheal intubation, propofol 1% was titrated through an infusion pump (Model OT-601, JMS, and Hiroshima, Japan), in order to maintain BIS between 40–50 throughout the surgical procedure. In other words, propofol was started with a dose of 100 mcg/kg/min and thereafter it was titrated to maintain BIS between 40–50 throughout the surgical procedure. Lungs were ventilated with 40%/60% nitrous oxide in oxygen. Atracurium 0.6 mg/kg/hr were infused as the muscle relaxation agent. The average mean arterial blood pressure (MAP) assessed at three intervals - at the time the patient referred to the anesthesia clinic, after his/her arrival in the operating room, and before the injection of pretreatment medication- was taken as the baseline values. Inadequate analgesia was defined as more than 20% increase in MAP from the baseline values for more than three min in response to a surgical stimulus; bolus doses of fentanyl 0.75 µg/kg were prescribed for these patients. At the end of the study period, maintenance dose (or total dose exception to induction) requirements of propofol (mg kg-1 h-1) and fentanyl (mcg kg-1 h-1) were measured by dividing the total amount of the individual drug used for maintenance by duration of the operation period and patient’s weight in kilograms; it should be noted that administered at the time of induction were not considered. IV crystalloid based on compensatory volume expansion, maintenance, deficit, third space and tripled amount of blood loss was administered along with packed red blood cells in cases which their hematocrit levels were lower than 30. Hypotension was defined as a 20% decrease in MAP from the baseline level. The latter was treated by NS infusion, and phenylephrine 50 mcg IV, if required. Considering the previous studies in this field, a total of 32 patients (16 patients in each group) were required with 80% power, and at 0.05 level of significance (4). SPSS Ver. 15 was employed to analyze the data by repeated measurement, chi-square and t-test.

## 4. Results

Thirty-two patients enrolled in the study were divided into two equal groups. The mean age of the patients under study was 55.5 ± 6.47 years. There were no differences in the demographic data between the two groups (Table 1 ). There was no difference in BIS levels of the two groups in the awake phase (P value = 0.224). Requirement of propofol for maintenance of anesthesia, aiming at achieving BIS levels between 40 and 50, in the bupivacaine group was 2.79 ± 0.36 mg/kg/hr compared to 6.02 ± 0.44 mg/kg/hr in the control group. The difference was reported to be statistically significant (P value < 0.001). A statistically significant reduction in the fentanyl requirement was noted in the bupivacaine group (P value < 0.001), suggesting that patients in the control group received higher doses of the drug. Table 2 outlines the amount of medication prescribed in each group. Hypotension requiring treatment with phenylephrine was reported in only three patients in the bupivacaine group (9.4%). None of the control group patients required treatment for hypotension. There was, however, no significant difference between the frequency of hypotension between the two groups (P value = 0.113). Complications including backache, dural tap were not encountered in either group under study.

**Table 1:** The Demographic Data of the Patients Enrolled in the Study

	Control	Bupivacaine	Total
**Age, y, Mean ± SD**	54.44 ± 6.73	56.56 ± 6.21	55.5 ± 6.47
**Gender, Male/Female**	9/7	9/7	18/14
**ASA Group, No. (%)**			
I	4 (25)	6 (37.5)	10 (31.2)
II	12 (75)	10 (62.5)	22 (68.7)
**MAP, mmHg, Mean ± SD**	86.25 ± 3.89	85.94 ± 3.99	86.09 ± 3.88

Abbreviations: ASA, the american society of anesthesiologists; MAP, mean arterial pressure

**Table 2:** The Dosage of Medication Prescribed in Each Group

	Control	Bupivacaine	CI 95%	P value
**Propofol maintenance, mg/kg/hr, Mean ± SD**	6.02 ± 0.44	2.79 ± 0.36	(-3.5)-(-2.9)	< 0.001
**Fentanyl, μg/kg/hr, Mean ± SD**	1.08 ± 0.21	0.11 ± 0.15	(-1.1)-(-0.8)	< 0.001

## 5. Discussion

The bispectral index score (BIS) has been introduced as an estimation of hypnotic effect (11). Previous studies have reported that BIS is a useful tool in predicting the adequacy of the depth of anesthesia during an operation regardless of the used anesthetic agent (12-14). Shono et al. compared the effects of epidural anesthesia with lidocaine 1% and 2% on hemodynamic variables and sevoflurane requirements during lower abdominal surgery under CGEA with BIS of 40-50; they reported that lidocaine 2% was more effective in reducing sevofluran’s requirement (15). Another study compared different doses of epidural ropivacaine (0.2% and 1%) on the propofol need during general anesthesia. It also revealed that higher doses of the medication were more effective in lowering the need for the anesthetic agent (8). Another study also reported that compared to high doses of bupivacaine, lower doses of the drug (0.625%) plus fentanyl (2µg/cc) have similar effects in reducing anesthetic need during general anesthesia (2). Agarwal et al. showed that epidural bupivacaine can reduce the dose requirement of propofol, fentanyl and vecuronium during general anesthesia (10). In their study, a significant reduction in the dose requirement of propofol for induction and maintenance of anesthesia was reported. The study also showed that the technique lowered the maintenance doses of fentanyl and vecuronium in the patients under study. In the same way, the current study showed that epidural bupivacaine substantially reduces the propofol requirement to achieve a predictable depth of anesthesia during the maintenance period. Similar to Agarwal et al., in the current study fentanyl was used to suppress autonomic reflexes due to noxious stimulus and increased levels of MAP (10). Fentanyl was administered to suppress these reflexes and, at the same time, propofol infusion was adjusted to maintain BIS within the range of 40–50. Researchers believe that CGEA reduces the need for general anesthetic agents; the underlying mechanism, however, is not completely understood. While some believe the direct influence of CGEA on the brain is independent of LA plasma levels, others claim that the blocking effect on the nociceptive input which originates from the surgical site contributes to the lowered need for general anesthetic agents in such individuals. Additionally, other studies maintain that supraspinal effects of the epidural analgesics, which suppress the individual’s level of consciousness, can reduce the need for general anesthetic agents (1). The afferentation theory also proposes that tonic sensory and muscles-spindle activity maintains a state of wakefulness (16, 17). It should be noted that Agarwal et al. used ephedrine to treat possible hypotension (10); previous studies, however, had reported the effect of this drug on the depth of anesthesia and the BIS levels during CGEA (18). Considering these facts, ephedrine was replaced with phenylephrine in the present study. It is noteworthy that compared to previous studies in which epidural agent was injected in the upper lumbar intravertebral areas (L2-L3 and L3-L4), in the current study the agent was injected into L4-L5 area and it was concluded that epidural anesthesia can reduce the anesthetic dose requirement even if lower areas are blocked. On the other hand, Ishiyama et al. reported that epidural anesthesia with ropivacaine reduces the BIS during the awake phase and the general anesthesia. Its effects, however, was reported to be more prominent in the latter period as physical, tactile, auditory, and visual stimulation such as BP measurement, electrocardiogram monitor’s sound, and the conversation among operating room staff may neutralize the sedative effects of epidural anesthesia during the awake phase (7). Performing CGEA using bupivacaine was reported to reduce propofol and fentanyl doses required to maintain BIS levels between 40 and 50 considerably. Limitation of the current study was that the blood concentration of the used medication and stress hormones were not assessed in the present study. It is possible that providing such measurements help anesthetic agents’ doses adjustment during the operation in order to achieve the satisfactory BIS; it may also rule out the belief that higher blood levels of local anesthetics lowers the BIS values. Epidural anesthesia using bupivacaine lowers the need for the anesthetic agents during CGEA without reducing the BIS levels in the awake phase. It is highly recommended to conduct larger studies, while using lower doses of local anesthetics and assessing their blood concentrations, in order to attain the lowest optimal dosage of combined epidural local anesthetics and general anesthetics, required for achieving the acceptable anesthesia in the presence of the least hemodynamic complications particularly in elderly patients and those suffering from cardiovascular diseases.
